# Telitacicept for the treatment of Sjögren’s disease complicated by unilateral vocal fold immobility, severe hypergammaglobulinemia, and diabetes: a case report

**DOI:** 10.3389/fimmu.2026.1956268

**Published:** 2026-10-14

**Authors:** Kedi Zheng, Zexuan Zhou, Guangzhou Du, Qisheng Lin, Jianqun Lin, Weijin Zhang, Shaoyu Zheng, Shijian Hu, Yukai Wang

**Affiliations:** 1 Department of Rheumatology and Immunology, Shantou Central Hospital, Shantou, Guangdong, China; 2 Department of Radiology, Shantou Central Hospital, Shantou, Guangdong, China

**Keywords:** ESSDAI score, hypergammaglobulinemia, Sjögren’s disease, telitacicept, type 2 diabetes mellitus, vocal fold immobility

## Abstract

Sjögren’s Disease (SjD) involves diverse extraglandular manifestations (EGMs). Severe hypergammaglobulinemia and suspected recurrent laryngeal nerve (RLN) -related unilateral vocal fold immobility represent aggressive phenotypes. Management of such presentations may be constrained by metabolic comorbidities such as diabetes, which limit the use of conventional high-dose glucocorticoids. Herein, we describe a 48-year-old female with SjD and Type 2 Diabetes Mellitus (T2DM) who presented with severe hypergammaglobulinemia (IgG 38.9 g/L), acute hoarseness, and sicca symptoms. Laryngostroboscopy demonstrated left-sided unilateral vocal fold immobility, possibly suggestive of neurogenic paralysis. Positron emission tomography-computed tomography (PET-CT) did not identify obvious masses or extrinsic compression along the visualized RLN course, reducing the likelihood of a compressive or malignant etiology. The EULAR Sjögren’s Syndrome Disease Activity Index (ESSDAI) score was 26. A combined therapeutic regimen comprising telitacicept (160 mg/week), methylprednisolone (20 mg/day) and hydroxychloroquine (0.2 g, twice daily) was initiated, both to minimize glucocorticoid exposure and to target the patient’s profound B-cell hyperactivity manifested by severe hypergammaglobulinemia. Marked serological and symptomatic improvements were observed during treatment. Within 15 weeks, the IgG level decreased to 14.6 g/L, rheumatoid factor (RF) decreased from 759 IU/mL to 58.3 IU/mL, and the ESSDAI score dropped to 6. The patient experienced symptomatic improvement of hoarseness, while methylprednisolone was successfully tapered to 4 mg/day. Clinical stability based on patient-reported symptoms persisted through the 12-month follow-up. This case is hypothesis-generating: A telitacicept-containing regimen may produce marked serological improvement and symptomatic benefit for high-activity SjD with laryngeal/vocal fold involvement, while reducing glucocorticoid-related metabolic risks. Definitive efficacy requires confirmation in larger controlled studies.

## 1 Introduction

Sjögren’s disease (SjD) is an autoimmune disease classically defined by exocrine gland infiltration and sicca symptoms (1). However, the clinical burden is largely driven by systemic extraglandular manifestations (EGMs), involving neurological, hematological, and visceral systems, which significantly determine prognosis and quality of life (2). Hypergammaglobulinemia, present in nearly half of SjD patients, serves as a critical marker of B-cell hyperactivity; it predisposes patients to severe visceral involvement and is independently associated with increased mortality (3). In the context of such high systemic disease activity, neurological sequelae can occur; while peripheral neuropathy is a well-recognized EGM, cranial nerve involvement-specifically affecting the recurrent laryngeal nerve (RLN), remains rare and poses both diagnostic and therapeutic challenges (4). Managing such aggressive phenotypes is further complicated in patients with metabolic comorbidities like diabetes mellitus, in whom the cumulative toxicity of high-dose glucocorticoids precludes standard immunosuppressive regimens. Telitacicept, a novel fusion protein targeting both B-lymphocyte stimulator (BLyS/BAFF) and a proliferation-inducing ligand (APRIL), offers a promising steroid-sparing strategy by inhibiting plasma cell survival (5). Herein, we report a case of SjD presenting with unilateral vocal fold immobility and severe hypergammaglobulinemia. After combination therapy comprising telitacicept, methylprednisolone and hydroxychloroquine, the patient experienced hoarseness resolution and marked serological improvement.

## 2 Case presentation

A 48-year-old female with chronic sicca symptoms (xerostomia and xerophthalmia) was diagnosed with SjD 10 years ago based on autoantibodies and salivary-gland histology. After a six-month course of glucocorticoids and hydroxychloroquine (doses undocumented), she improved clinically but discontinued treatment without medical advice. She had a comorbid history of Type 2 Diabetes Mellitus (T2DM), with fasting blood glucose fluctuating between 7 and 8 mmol/L. In June 2023, she developed generalized pruritic rash, fatigue, dry cough, polyarthralgia with morning stiffness and anorexia; acute hoarseness followed in September, and she presented to our hospital on October 16, 2023.

Admission examination revealed a fatigued state, 10-kg weight loss, and widespread reddish-brown maculopapular eruptions on the extremities clinically suggestive of cutaneous vasculitis, but the patient declined skin biopsy. Pulmonary auscultation noted moist rales. Laryngostroboscopy (**Figure 1**) demonstrated left-sided vocal fold immobility: during inspiration, the left vocal cord and arytenoid cartilage remained fixed in the paramedian position, while the right vocal cord abducted normally. During phonation, the left vocal fold failed to adduct toward midline, producing incomplete glottic closure and total loss of the mucosal wave. These findings are consistent with neurogenic-type vocal fold immobility; however, laryngostroboscopy alone cannot differentiate neurogenic paralysis from mechanical fixation such as cricoarytenoid joint disease.

**Figure 1:**
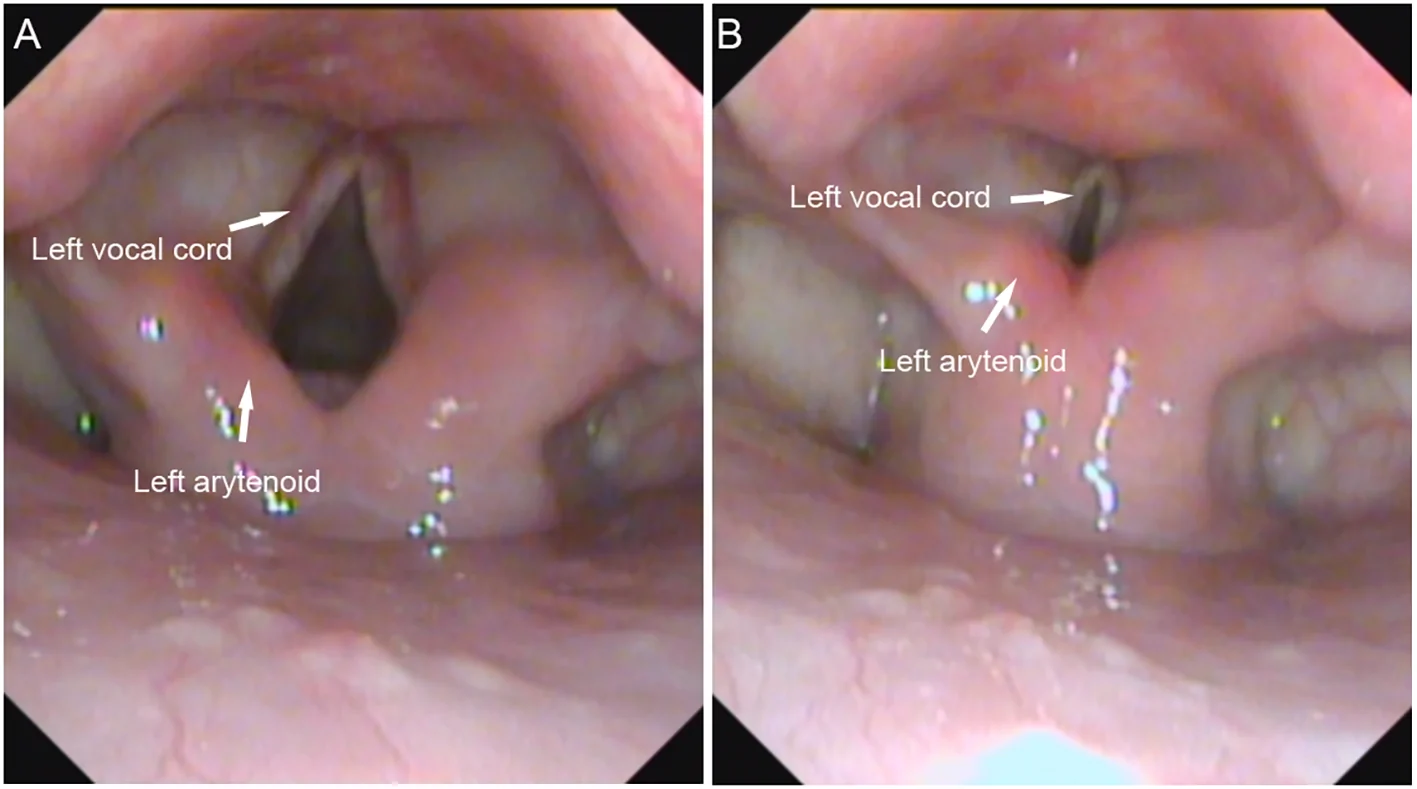
Baseline laryngostroboscopy findings. **(A)** Inspiratory phase: The left vocal cord and arytenoid cartilage (arrows) remain fixed in a paramedian position while the contralateral side abducts normally, resulting in a narrowed glottic aperture. **(B)** Phonatory phase: Upon attempted phonation, the left vocal cord fails to adduct to the midline, leading to incomplete glottic closure and a persistent glottic gap. Dynamic assessment further revealed a complete loss of the mucosal wave on the left side, suggesting the possibility of neurogenic paralysis. This finding cannot exclude mechanical fixation such as cricoarytenoid joint disease.

Investigations indicated severe systemic inflammation and robust B-cell hyperactivation: antinuclear antibody (ANA) 1:3200, positive anti-Sjögren’s syndrome-related antigen A (anti-SSA)/Ro52 antibody, and a markedly elevated rheumatoid factor (RF) of 759 IU/mL (Normal range [NR]: 0–20 IU/mL) (**Table 1**). Profound hypergammaglobulinemia was observed: IgG 38.9 g/L (NR: 7.0-16.0 g/L), globulin 55.5 g/L (NR: 20.0–35.0 g/L). Complement 3 (1.2 g/L) and complement 4 (0.2 g/L) levels were within normal limits. Glycated hemoglobin was 9%. Serum immunofixation electrophoresis excluded abnormal monoclonal immunoglobulin bands. Urine light-chain assay showed κ-light chain 160 mg/L (NR: 0–18.5 mg/L) and λ-light chain < 50 mg/L (NR: 0–50 mg/L). Peripheral-blood lymphocyte-subset analysis yielded the following results: CD3^+^ total T-lymphocytes 83.41 %, CD4^+^ T-cells 41.79 %, CD8^+^ T-cells 40.37 %, CD4/CD8 ratio 1.04 , CD3-CD19^+^ B-lymphocytes 13.19 %, CD3-CD16^+^CD56^+^ NK-cells 1.77 %.

**Table 1:** Longitudinal lab data.

Parameters	Baseline 2023-10-16	1 month 2023-11-10	3 months 2024-01-27	12 months 2024-09-29 (telitacicept discontinued)
Hematology				
Hb, g/L	103	101	115	104
Immunoglobulins				
IgG, g/L	38.9	23.3	14.6	22.7
IgA, g/L	3.71	2.23	0.74	2.45
IgM, g/L	0.98	0.62	0.33	0.529
Serology & Inflammation				
RF, IU/mL	759	207	58.3	32
ESR, mm/h	62	99	56	83
CRP, mg/L	4.64	7.24	4.8	8.42
C3, g/L	1.2	1.36	1.27	1.25
C4, g/L	0.2	0.25	0.28	0.321
Biochemistry				
Albumin, g/L	32.9	35.7	42.3	37.7
Globulin, g/L	55.5	41.5	31.9	44.1
Disease Activity				
ESSDAI Score	26	16	6	–
ESSPRI Score	5.33	2	1.67	–

Hb, Hemoglobin; IgG, Immunoglobulin G; IgA, Immunoglobulin A; IgM, Immunoglobulin M; RF, Rheumatoid Factor; ESR, Erythrocyte Sedimentation Rate; CRP, C-reactive Protein; C3, Complement 3; C4, Complement 4; ESSDAI, EULAR Sjögren’s Syndrome Disease Activity Index; ESSPRI, EULAR Sjögren’s Syndrome Patient Reported Index.

Brain CT and whole-body PET-CT imaging were completed (**Figure 2**). Brain CT showed no obvious intracranial space-occupying lesions. PET-CT demonstrated no definite mass or extrinsic compression along the cervical-mediastinal course of the RLN, reducing the likelihood of malignant disease. Multiple small mediastinal lymph nodes showed mildly increased FDG uptake (largest short-axis diameter 0.4 cm, SUVmax 1.8) and bilateral axillary small lymph nodes also presented mild FDG hypermetabolism (maximal short-axis 0.6 cm, SUVmax 1.3). These imaging features favored reactive lymphadenopathy.

**Figure 2:**
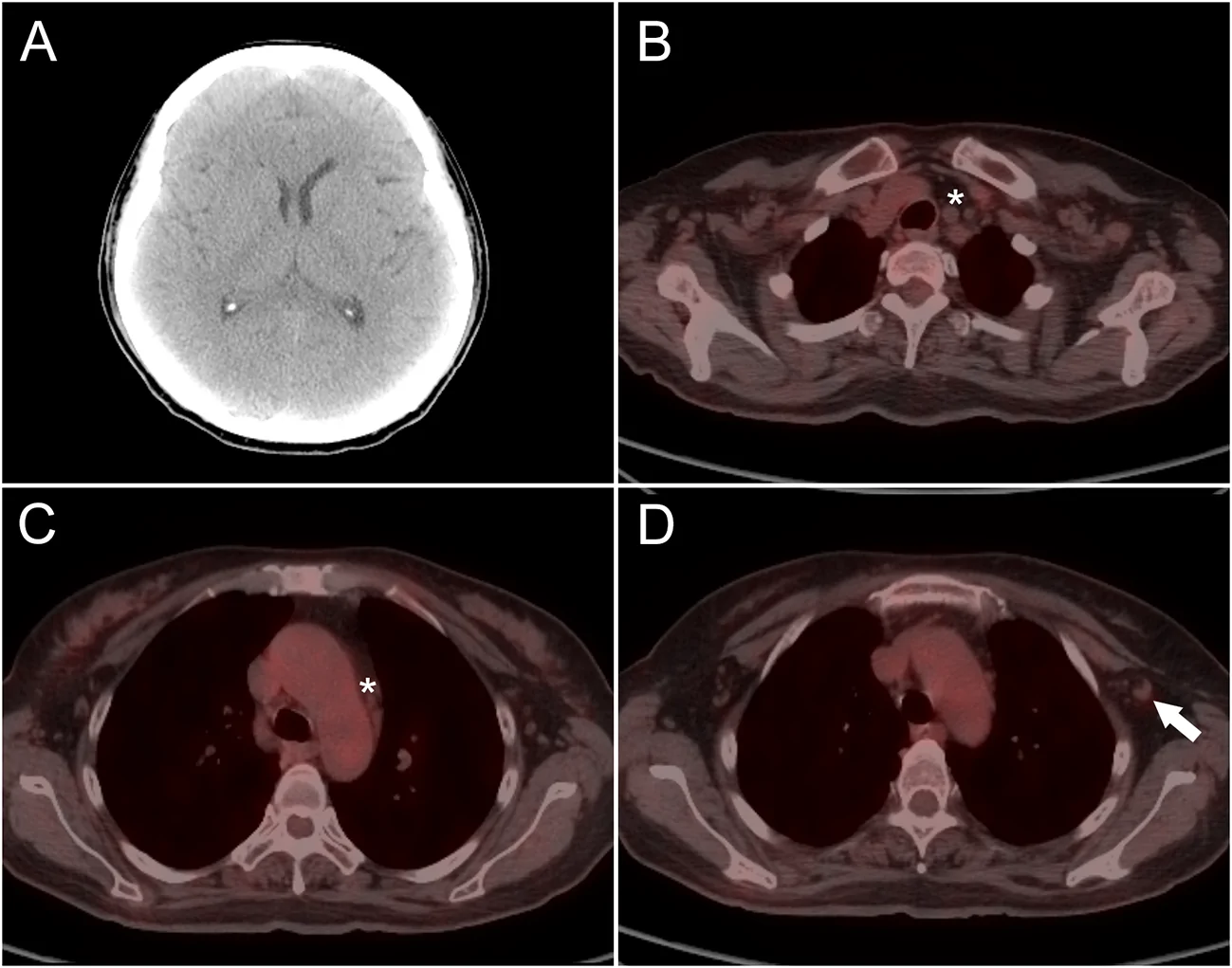
PET-CT Images. **(A)** Brain CT scan demonstrating no evidence of intracranial hemorrhage or space-occupying lesions; note that brain CT is limited for evaluation of brain-stem and skull-base lesions. **(B)** Transaxial PET-CT image showing no obvious mass compression along the cervical course of the recurrent laryngeal nerve (indicated by asterisk). **(C)** Transaxial PET-CT image showing no obvious mass compression along the mediastinal course of the recurrent laryngeal nerve (indicated by asterisk). **(D)** Enlarged left axillary lymph node (white arrow) with increased metabolic activity, consistent with reactive hyperplasia and systemic immune activation. Malignant pathology cannot be fully excluded by PET-CT alone.

At presentation, the ESSDAI score was 26 (constitutional 6, articular 2, cutaneous 9, pulmonary 5, hematological 2, biological 2), while the concurrent EULAR Sjögren’s Syndrome Patient Reported Index (ESSPRI) score was 5.33, suggestive of active SjD.

Given the patient’s high disease activity, profound hypergammaglobulinemia and suspected cranial-nerve involvement, an aggressive therapeutic strategy was warranted. Considering concomitant T2DM, prolonged high-dose glucocorticoid exposure was avoided to mitigate substantial metabolic and infectious risks. Telitacicept was selected for its dual-blockade of BLyS/APRIL, which targets both mature B cells and pathogenic plasma cells to counteract marked B-cell hyperactivation; phase 2 trial data in primary Sjögren’s syndrome demonstrate its capacity to reduce disease activity and immunoglobulin levels, rendering it a potential steroid-sparing agent (5). However, telitacicept had not yet received National Medical Products Administration (NMPA) approval for Sjögren’s disease; hence it was administered off-label. Written informed consent was obtained after thorough patient counseling. The therapeutic regimen combined subcutaneous telitacicept 160 mg/week, methylprednisolone 20 mg/day and hydroxychloroquine 0.2 g twice daily.

Noticeable clinical and laboratory improvements were observed. After one month of therapy, IgG decreased to 23.3 g/L and RF decreased to 207 IU/mL (**Figure 3**). Marked serological and clinical improvement was evident at the 3-month follow-up: vasculitic rash had resolved, and the patient reported subjective improvement in hoarseness, polyarthralgia, dry cough and sicca symptoms. The ESSDAI score dropped from 26 to 6. IgG levels reached a nadir of 14.6 g/L, RF fell to 58.3 IU/mL, and serum albumin levels normalized from 32.9 g/L to 42.3 g/L.

**Figure 3:**
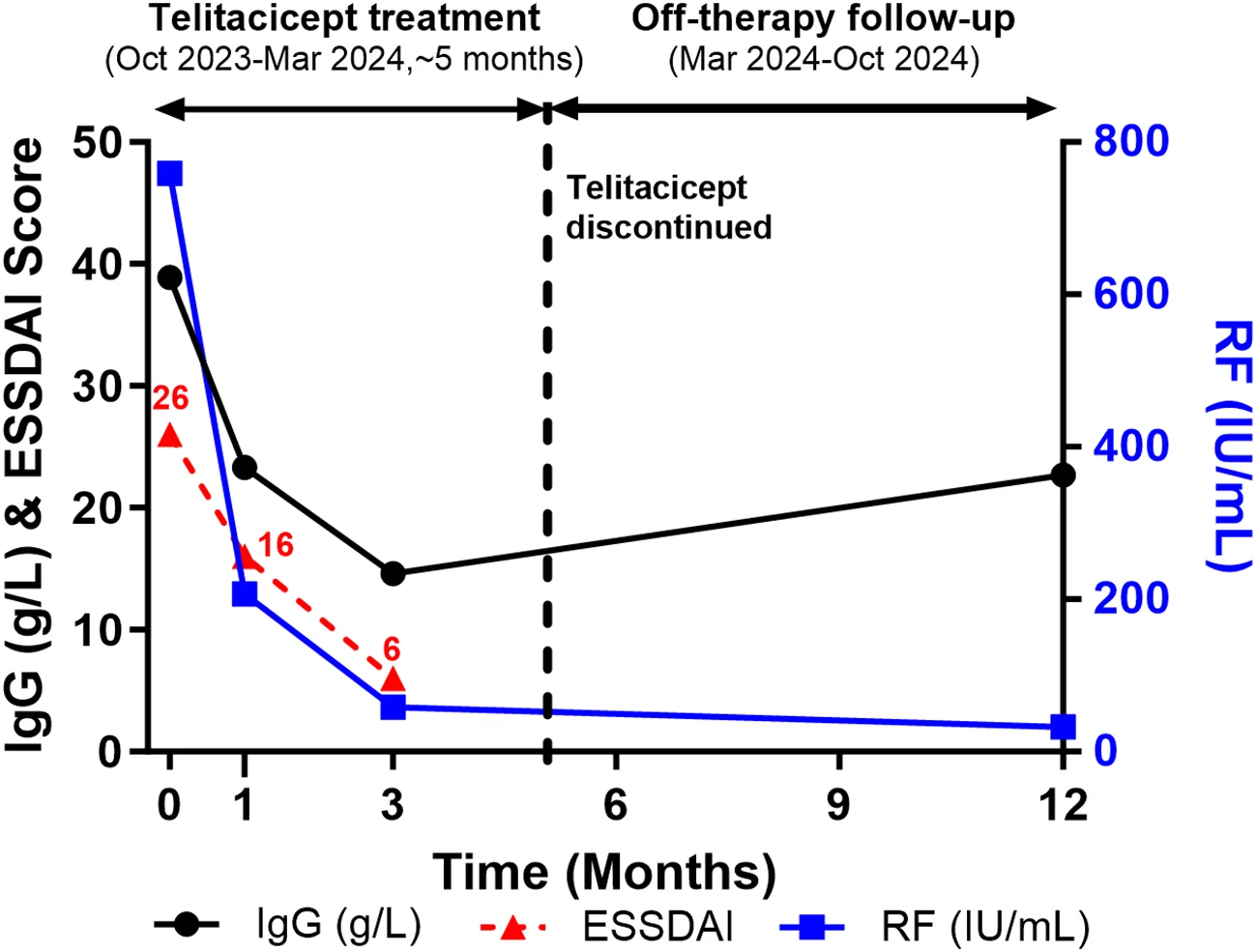
Longitudinal trajectories of clinical disease activity and serological markers. Serum IgG (black circles), rheumatoid factor (RF; blue squares), and ESSDAI scores (red triangles) are illustrated over a 12-month period. The left Y-axis corresponds to serum IgG levels (g/L) and ESSDAI scores, while the right Y-axis corresponds to RF titers (IU/mL). ESSDAI values are 26 at baseline, 16 at month 1, and 6 at month 3. ESSDAI was not assessed at the final 12-month visit. Telitacicept treatment period spanned October 2023-March 2024. The vertical dashed line marks telitacicept discontinuation, followed by off-therapy follow-up until month 12.

Methylprednisolone was tapered to 4 mg daily at month 3. Complete glucocorticoid withdrawal was deferred owing to substantial prior systemic disease burden and suspected cranial-nerve involvement. Telitacicept was discontinued in March 2024 due to financial constraints related to its off-label use. At the 12-month follow-up visit, the patient remained clinically stable on symptom-based evaluation, with sustained improvement of hoarseness, no vasculitis relapse, persistent relief of sicca symptoms and absence of new systemic manifestations. No infectious complications, new-onset or treatment-worsening cytopenia, clinically significant hypogammaglobulinemia or other drug-related adverse events occurred during observation. Fasting blood glucose fluctuated between 6–8 mmol/L throughout follow-up.

## 3 Discussion

This case illustrates diagnostic and therapeutic challenges in SjD presenting with unilateral vocal fold immobility, severe hypergammaglobulinemia and comorbid T2DM. A steroid-sparing approach was preferable given glucocorticoid-related metabolic risks, and clinical and serological improvement was achieved with the combination regimen of telitacicept, methylprednisolone and hydroxychloroquine.

Unilateral vocal fold immobility is a rare extraglandular manifestation of SjD. Potential mechanisms include immune-mediated RLN neuropathy, small-vessel vasculitis of the vasa nervorum, perineural lymphocytic infiltration, or cricoarytenoid joint involvement (6). Laryngostroboscopy alone cannot distinguish joint fixation from neurogenic paralysis (7). In this patient, the absence of laryngeal pain, arytenoid tenderness, trauma, intubation history, and cricoarytenoid FDG hypermetabolism made immune-mediated RLN involvement more likely.

Severe hypergammaglobulinemia and elevated RF indicate severe systemic disease and increased B-cell lymphoma risk (8). For patients with such profound B-cell hyperactivation, therapeutic priorities include adequate disease control while minimizing steroid-related harm in metabolically vulnerable individuals. As a dual BLyS/APRIL inhibitor, telitacicept targets both mature B cells and long-lived CD20-negative plasma cells, which are the primary drivers of hypergammaglobulinemia and autoantibody production (9). Phase 2 clinical data have supported its capacity to lower disease-activity indices and immunoglobulin levels in active SjD with an acceptable safety profile (5). In our index case, the substantial reduction of IgG and improvement in ESSDAI are broadly concordant with these trial-derived observations.

This complex real-world case differs from strictly selected clinical-trial populations but may inform treatment of SjD patients with severe hypergammaglobulinemia and suspected cranial-nerve involvement, suggesting telitacicept as a possible option. Additional high-quality evidence is still needed to confirm these observations.

Several limitations should be acknowledged. Skin biopsy, lymph-node biopsy, brain MRI and repeat laryngoscopy were not performed. Laryngeal electromyography was unavailable, precluding definitive differentiation between RLN palsy and cricoarytenoid joint disease. Furthermore, as methylprednisolone and hydroxychloroquine were administered concurrently, clinical benefit cannot be attributed solely to telitacicept, and spontaneous improvement of vocal fold immobility cannot be excluded.

In conclusion, telitacicept-containing combination therapy may improve serological and clinical outcomes in high-activity SjD while reducing glucocorticoid exposure in metabolically vulnerable patients. Definitive efficacy conclusions require large randomized controlled trials.

## 4 Patient perspective

The patient reported improvement in xerostomia and xerophthalmia, reduced fatigue, regression of the skin lesions, and improvement in hoarseness after treatment. She perceived the overall treatment effect as favorable.

## Data Availability

The raw data supporting the conclusions of this article will be made available by the authors, without undue reservation.
